# The Health Impact of Surgical Techniques and Assistive Methods Used in Cesarean Deliveries: A Systemic Review

**DOI:** 10.3390/ijerph17186894

**Published:** 2020-09-21

**Authors:** Li-Hsuan Wang, Kok-Min Seow, Li-Ru Chen, Kuo-Hu Chen

**Affiliations:** 1 Department of Obstetrics and Gynecology, Taipei Tzu-Chi Hospital, The Buddhist Tzu-Chi Medical Foundation, Taipei 231, Taiwan; 100311023@gms.tcu.edu.tw; 2 Department of Obstetrics and Gynecology, Shin Kong Wu Ho-Su Memorial Hospital, Taipei 111, Taiwan; M002249@ms.skh.org.tw; 3 Department of Obstetrics and Gynecology, National Yang-Ming University, Taipei 112, Taiwan; 4 Department of Physical Medicine and Rehabilitation, Mackay Memorial Hospital, Taipei 10449, Taiwan; gracealex168@gmail.com; 5 Department of Mechanical Engineering, National Chiao-Tung University, Hsinchu 30010, Taiwan; 6 School of Medicine, Tzu-Chi University, Hualien 970, Taiwan

**Keywords:** cesarean delivery, uterine exteriorization, in situ repair, vacuum, fundal pressure

## Abstract

Cesarean delivery is one of the most frequently performed surgeries in women throughout the world. However, the most optimal technique to minimize maternal and fetal morbidities is still being debated due to various clinical situations and surgeons’ preferences. The contentious topics are the use of vacuum devices other than traditional fundal pressure to assist in the delivery of the fetal head and the techniques of uterine repair used during cesarean deliveries. There are two well-described techniques for suturing the uterus: The uterus can be repaired either temporarily exteriorized (out of abdominal cavity) or in situ (within the peritoneal cavity). Numerous studies have attempted to compare these two techniques in different aspects, including operative time, blood loss, and maternal and fetal outcomes. This review provides an overview of the assistive method of vacuum devices compared with fundal pressure, and the two surgical techniques for uterine repair following cesarean delivery. This descriptive literature review was performed to address important issues for clinical practitioners. It aims to compare the advantages and disadvantages of the assistive methods and surgical techniques used in cesarean deliveries. All of the articles were retrieved from the databases Medline and PubMed using the search terms cesarean delivery, vacuum, and exteriorization. The searching results revealed that after exclusion, there were 9 and 13 eligible articles for vacuum assisted cesarean delivery and uterine exteriorization, respectively. Although several studies have concluded vacuum assistance for fetal extraction as a simple, effective, and beneficial method during fetal head delivery during cesarean delivery, further research is still required to clarify the safety of vacuum assistance. In general, compared to the use of in situ uterine repairs during cesarean delivery, uterine exteriorization for repairs may have benefits of less blood loss and shorter operative time. However, it may also carry a higher risk of intraoperative complications such as nausea and vomiting, uterine atony, and a longer time to the return of bowel function. Clinicians should consider these factors during shared decision-making with their pregnant patients to determine the most suitable techniques for cesarean deliveries.

## 1. Introduction

Cesarean delivery is one of the most frequently performed operative procedures in women throughout the world, and the rate of surgery is still increasing. Since 1985, the international healthcare community has considered the ideal rate for cesarean sections to be between 10% and 15% [1]. According to the report in the literature, the global prevalence rate of cesarean delivery is estimated to be around 15% (15.9% in Asia, 19% in Europe and 3.5% in Africa) [2]. However, cesarean sections have become increasingly common in both developed and developing countries [3]. For instance, the rate of cesarean delivery in the United States rose from 26% in 2003 to 36.5% in 2009 [1]. This phenomenon is noted in various countries worldwide [4,5].

Although cesarean delivery is a common surgery, the most optimal technique to minimize maternal and fetal morbidities is still being debated due to various clinical situations and surgeons’ preferences. One of the concerns is the assistive methods used to facilitate the delivery of the fetal head once the uterine incision is made. The traditional method is to push the fetus out of the incision by manually applying pressure on the fundus of the uterus. Alternatively, the fetal head can be pulled out by a vacuum device, which is applied to the fetal head. Currently, there are few studies that explore the benefits and disadvantages of vacuum application during cesarean delivery. Another contentious topic is the methods of uterine repair following cesarean deliveries. There are two well-described techniques for suturing the uterus: The uterus can be temporarily exteriorized out of the abdominal cavity for repairs, or it may remain in situ within the peritoneal cavity. Numerous studies have attempted to compare these two techniques in different aspects, including operative time, blood loss, and maternal and fetal outcomes. Nonetheless, the study results are inconsistent and do not address all issues and concerns.

This review provides an overview of these methods, including the use of an assistive vacuum device or fundal pressure for the delivery of the fetal head and two techniques for uterine repair following cesarean delivery. It aims to compare the advantages and disadvantages of the assistive methods (the vacuum device vs. fundal pressure) and surgical techniques (uterine exteriorization vs. in situ repair) used in cesarean deliveries after searching the databases Ovid Medline and PubMed. This descriptive literature review was performed to address important issues for clinical practitioners.

## 2. Technique Description

Cesarean delivery (formerly called a cesarean section) is one of the major surgical procedures performed worldwide and accounts for almost one-third of births in the United States [6,7]. According to the recommendation of the World Health Organization (WHO) Statement on Cesarean Section Rates, the ideal rate for cesarean sections was modified from 10% to 15% in 1985. However, cesarean deliveries have continued to increase in countries with and without high per capita incomes [1]. A previous study by Boerma et al. reported that the global cesarean section rate increased from 12.1% in 2000 to 21.1% of all births in 2015 [8].

For cesarean delivery, there are no standard procedures or best techniques. Still, evidence-based recommendations for surgical techniques are suggested in previous articles among which, the advantages and disadvantages of various incisions and procedures during the operation, as performed in recent years, could be compared [9]. The surgical procedures during cesarean delivery include the skin incision and the incision from the subcutaneous tissue layer to the fascial and rectus muscle layers. Following these steps, the peritoneum is opened, and then, the hysterotomy is performed. Subsequently, fetal and placental extraction from the incision of the uterus is followed by uterine repair. Finally, the abdominal wall to the skin is closed by layer. As with other open abdominal surgeries, several of these steps are still under discussion. Some randomized controlled trials mention variations of these surgical techniques; both the advantages and disadvantages are evaluated [10,11,12,13,14].

For instance, transverse incisions provide better cosmetic appearance postoperatively. However, a midline vertical incision could be made in an emergency or to provide better exposure of the operative field. Seiler et al. stated that there were no significant differences in 30-day mortality, one-year mortality, pulmonary complications, hospitalization days, or hernias between midline and transverse incisions. Wound infections occurred more frequently in the transverse incision group [15]. In addition, many articles compared the two different ways of making transverse incisions for cesarean delivery. Similar findings have been reported for the Joel-Cohen type incision compared to the Pfannenstiel incision, which revealed significantly better results in the short-term, such as lower rates of fever, less blood loss, less postoperative pain, and shorter durations of procedures and hospital stays [14,16].

After the fetus is delivered, the wound made by the uterine incision is sutured after the placenta is extracted. We focused on the comparison of the assistive methods to facilitate the delivery of the fetal head and the two different choices for uterine repair, either uterine exteriorization or in situ repair. Intra-abdominal repair during cesarean delivery means that the uterine incision would be sutured in situ. Alternatively, extra-abdominal repair means that the uterine incision is sutured after uterine exteriorization and manual delivery from the uterine fundus, which temporarily provides access to the patient’s abdomen and pelvis before closing the uterus. Externalizing the uterine fundus helps to facilitate uterine massage effectively and easily. It also facilitates the examination of the hysterotomy site and adnexa efficiently by providing a better surgical field of view. The uterus is then returned to the abdominal cavity before the abdominal wall repair [17]. 

Both intraoperative complications, such as nausea, vomiting, and abdominal pain, and postoperative complications, including infections, hemorrhage, and visceral injury, are important considerations [18]. Infections have been noted to occur in up to 10% patients after cesarean delivery, even when prophylactic antibiotics are used [18]. Rehospitalization after discharge, most commonly for uterine infections or hemorrhage, is more common after cesarean than vaginal deliveries, although the difference is less than 1 per 100 discharges [18]. In addition, the effect of adhesion formation after cesarean delivery on subsequent abdominal surgery and its potential for causing infertility need to be evaluated further [18].

Nevertheless, the technique of uterine exteriorization repair carries the risk of pelvic adhesions between the peritoneum, uterus, and bowels due to possible abrasion of the uterus during the uterine exteriorization [19,20]. Postoperative adhesions are a natural consequence of surgical tissue trauma and healing [19,20]. Consequences of peritoneal adhesions include infertility, bowel obstruction, or abdominal/pelvic pain and may increase the technical difficulty of subsequent abdominal or pelvic surgeries [19,20]. Another potential risk of the technique is intraoperative nausea and vomiting due to poor blood perfusion when the uterus is everted outside the abdominal cavity and exposed to room air.

There have been several randomized controlled trials and meta-analyses that have compared exteriorized uterine repairs with in situ (non-exteriorization or intra-abdominal) uterine repairs. In this review, we discuss the differences in the rates of intraoperative and postoperative complications and morbidities and the advantages and disadvantages of the techniques [21,22].

## 3. Materials and Methods

This review was modeled based on the Preferred Reporting Items for Systematic Reviews and Meta-Analyses (PRISMA) statement. All of the articles were retrieved from the databases Medline and PubMed using the search terms “cesarean delivery” and “vacuum” for the assistive method “vacuum assisted cesarean delivery”, and the search terms “cesarean delivery” and “exteriorization” for the surgical technique “uterine exteriorization”. For further screening and selection, only full-text articles were considered to be included for a further analysis. 

A literature search was conducted on Medline and PubMed databases up to 31 May 2020 to identify all potential articles in the data sources. In the screening process, non-human research, duplication articles and research before 1985 were excluded. After initial processing, two experts in the field independently reviewed potentially eligible studies for exclusion and inclusion. Articles with poor study design or not-matched outcomes were deemed not eligible for the study. During the period of study selection, disagreements between the two reviewers were resolved by mutual discussion until a consensus was reached.

## 4. Search Results

**Figure 1:**
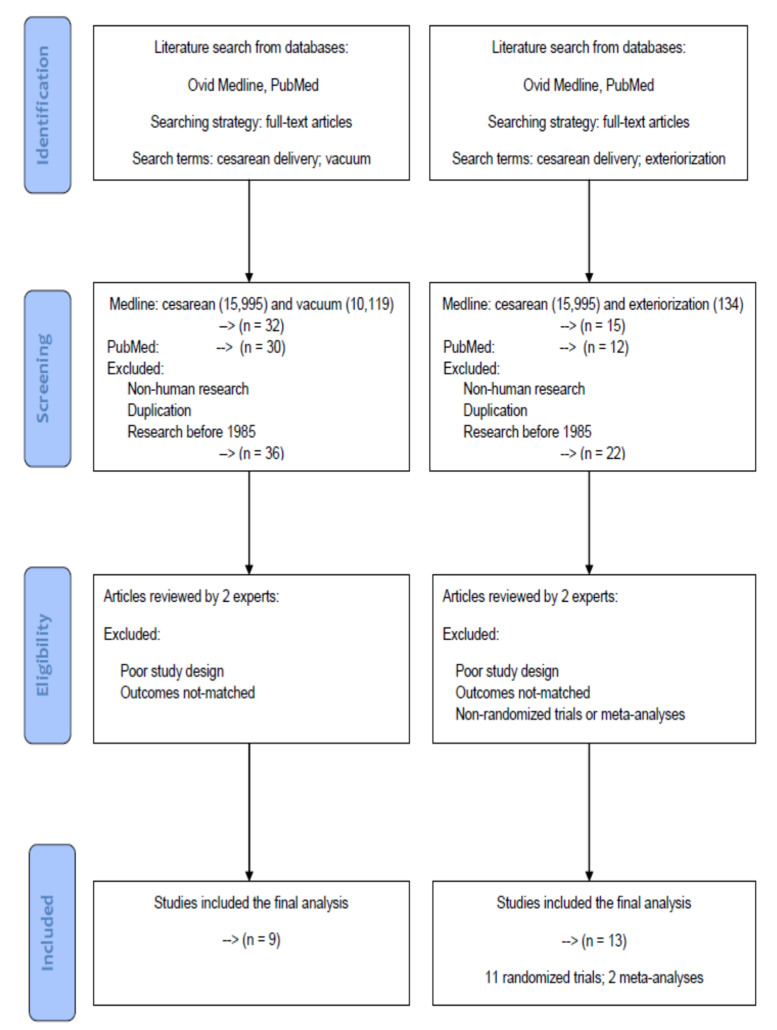
A flowchart of article selection to illustrate the processes of database identification, article screening, consideration of eligibility, and final inclusion according to the PRISMA statement.

A flowchart of article selection has been shown in Figure 1 to illustrate the processes of database identification, article screening, consideration of eligibility and final inclusion according to the PRISMA statement. Using the search terms and strategy mentioned above, there were 32 (Medline) and 30 (PubMed) articles in the assistive method “vacuum assisted cesarean delivery”, as well as 15 (Medline) and 12 (PubMed) articles in the surgical technique “uterine exteriorization”. After the screening process to exclude non-human research, duplication articles, and research before 1985, 36 and 22 articles were considered for further analysis.

Each article was evaluated and extracted independently by two reviewers to inspect the sources including year, chosen types of assistive methods or surgical techniques, research design, and clinical outcomes. Differences in selection between the two reviewers were reassessed and discussed until a consensus was reached. After excluding articles with poor study design or not-matched outcomes, there were 9 and 13 articles for the assistive method “vacuum assisted cesarean delivery”, and the surgical technique “uterine exteriorization”, respectively. There were 11 randomized trials and 2 meta-analyses eligible for further comparisons between the uterine exteriorization and in situ groups.

## 5. Surgical Techniques and Assistive Devices Used in Cesarean Deliveries

### 5.1. Vacuum-Assisted Cesarean Delivery

#### 5.1.1. Definitions

Difficult cesarean deliveries may bring about fetal injuries or morbidities. In previous studies researching assistive methods during cesarean deliveries, vacuum assistance was compared with manual delivery using fundal pressure to facilitate the delivery of a baby [23].

Vacuum-assisted cesarean delivery refers to the use of a vacuum device on the fetal head to facilitate the extraction of the fetus. According to the literature review, original vacuum extraction cups for delivering the fetus were made of rigid metal. Subsequently, soft cups of flexible materials such as silicone rubber or plastic were introduced [24]. Solomons et al. first published an article entitled “Malmstrom vacuum extractor during cesarean section” in 1962 and described the “Malmström cup” [25].

#### 5.1.2. Comparisons of Different Assistive Methods for Delivering the Fetal Head

McQuivey and Block reviewed the literature on vacuum-assisted cesarean deliveries before 2016 [26]. They believed the main advantages of using a vacuum device during a cesarean section was to assist with the delivery of the fetal head. First, by avoiding the use of a delivering hand or forceps blade on the fetal head, the area being delivered through the uterine incision could be decreased when the vacuum device is used correctly. Second, reducing the traumatic or damaging extension of the uterine incision and its associated complications such as excessive blood loss, vacuum assistance would be helpful. Finally, it was also used to decrease the amount of fundal pressure, which is necessary for the delivery of the fetal head. Thereby, it also reduced maternal discomfort at the same time [26].

Another benefit recounted by Bofill et al. was that patients reported less pain with the vacuum-assisted cesarean delivery, probably due to the reduction of fundal pressure, which was necessary for delivery [27]. Theoretically, excessive fundal pressure on the uterus may result in localized pain and even uterine rupture. Such complications can be avoided by “drawing the baby out” with a vacuum device.

Dimitrov et al. also concluded that the use of a vacuum extractor in cesarean deliveries was a rapid method that made surgeons abandon the need for rough and prolonged fundal compression. Moreover, it was proved that significantly less time was needed for scalp traction in the soft-cup vacuum extractor group than in the manual extraction group [28]. Not only was the total duration of time for the cesarean delivery reduced but also the total blood loss was lower in the vacuum-assisted group. However, the results in this prospective study showed no significant differences in the Apgar scores at the first or fifth minute for these newborns between the two groups [28].

Steven et al. discussed fetal injuries due to vacuum assistance during cesarean delivery in a case report in 2008 [29]. A 42-year-old woman scheduled for a cesarean delivery underwent the delivery with the assistance of a soft silastic vacuum device. A cephalohematoma and subgaleal hemorrhage had occurred in the newborn’s head, which was found after delivery. Moreover, cerebellar, subarachnoid, and intraparenchymal hemorrhages with mass effect resulting in ventriculomegaly were revealed with later imaging. Encephalomalacia, which was secondary to the infarction from the intracranial hemorrhage, subsequently happened. In the author’s opinion, clear evidence of benefits and safety for routine use of vacuum extraction during cesarean deliveries was still absent, and the procedure was not recommended to be performed routinely due to its potential for serious fetal injuries [29].

Arad et al. conducted a prospective study and demonstrated that in the regular cesarean section group, the interval between the final uterine incision and complete delivery was prolonged significantly (*p* < 0.01) compared with the vacuum extraction group during cesarean delivery [30]. However, the Apgar scores and acid-base values of the neonates in the two groups were not significantly different [30].

The benefits and suspected disadvantages of vacuum-assisted extraction during cesarean deliveries were listed in the above paragraphs. Most of these early articles reported the same results of no significant difference in the Apgar scores of newborns [28,30,31].

#### 5.1.3. Summary

In summary of the previous studies, most authors agreed that vacuum-assisted cesarean delivery was a safe, rapid, and effective skill, reducing the total duration of the deliveries and the need for prolonged fundal compression, which was used manually for extraction of the baby [26,27,28,30,31].

However, difficult vacuum deliveries sometimes resulted in fetal injury or complications such as cephalohematoma, subgaleal hemorrhage, neonatal depression, bruising of the fetal scalp, or the presence of a chignon [26,29,32].

Although several studies of cesarean deliveries have recognized vacuum assistance for fetal extraction as a simply equipped, effective, and beneficial method of fetal head delivery, further research is required to clarify the safety of vacuum assistance in cesarean deliveries.

### 5.2. Comparison of the Different Techniques of Uterine Repair during Cesarean Deliveries

In the following literature review, we compared two techniques (uterine exteriorization vs. in situ) of uterine repair during cesarean delivery. We examined intraoperative issues such as blood loss, nausea, vomiting, the length of the operation, and uterine atony. We also examined postoperative issues, including postoperative complications (pain, fever, endometritis, wound infection, and bowel dysfunction) and hospitalization days.

#### 5.2.1. Blood Loss

Minimization of blood loss during cesarean delivery is an important issue for all obstetricians because excessive blood loss has been recognized as a major cause of maternal morbidity and mortality [32]. Theoretically, the exteriorization of the uterus for uterine repair may help to reduce blood loss by kinking of the uterine arteries during exteriorization. Another possible mechanism is more effective bimanual compression of the uterus and better access to the uterine incision with faster suturing and hemostasis due to a relatively bloodless operative field [33,34]. Orji et al. reported a significant difference in mean estimated blood loss (459.8 mL in the exteriorized uterine repair group versus 546.8 mL in the in situ group) in a randomized controlled trial comparing the two uterine repair techniques [33]. Similar results were noted in another study by Ezechi et al. (742 mL vs. 872 mL) [34]. Moreover, the mean postoperative drop in hemoglobin level differed in several studies, ranging from 1–1.4 g/dL in the exteriorized uterine repair group and 1.2–1.7 g/dL in the in situ (non-exteriorization) repair group [35,36,37,38]. Despite significant differences in estimated blood loss and the postoperative drop in hemoglobin level, the percentage of blood transfusions after cesarean delivery between the two uterine repair techniques did not differ in previous studies [17,33,34,36,37,38,39]. A possible explanation could be that the difference in blood loss between the two uterine repair groups might have lacked a clinically significant impact. However, in the aspect of better control of blood loss and hemostasis, many surgeons still favored exteriorization for uterine repair.

#### 5.2.2. Intraoperative Complications

Intraoperative complications such as nausea, vomiting, and pain relate directly to patient comfort during cesarean delivery for women undergoing regional anesthesia [40,41]. Since many factors, especially operation and anesthesia techniques, may contribute to these complications, several studies investigating their association with uterine repair methods have been conducted. Siddiqui et al. reported that in women who received spinal anesthesia during cesarean delivery, a higher incidence of intraoperative nausea and vomiting was noted in the exteriorized uterine repair group (38%) than in the intra-abdominal repair group (18%) [42]. It was hypothesized that exteriorizing the uterus could lower mean arterial pressure, thus causing nausea and vomiting during continuous fundal pressure [43]. However, another randomized clinical trial and meta-analysis reported no statistically significant differences in intraoperative nausea, vomiting, or pain between the two methods of uterine repair [21]. Recently, a randomized controlled trial focused on the effects of uterine repair on intraoperative nausea and vomiting in 1120 women who underwent repeated cesarean delivery. The investigators found a significantly higher incidence of intraoperative nausea and vomiting in the exteriorized uterine repair group (38.7%) versus the in situ repair group (24%) [44]. Moreover, another randomized controlled trial published in May 2020 found that in situ uterine repairs during elective cesarean delivery was associated with less post-delivery intraoperative nausea and vomiting [45]. Nevertheless, there was no definite conclusion regarding this issue. More effort can be made to explore both the benefits and adverse effects between these two methods of uterine repair to aid in determining which technique to perform.

#### 5.2.3. Operative Time

Operation time is also an important consideration for both surgeons and patients. Most of the previous studies reported no significant difference in total operative time between the exteriorization and in situ techniques of uterine repair [9,33,38,42,43,46]. Nevertheless, other investigators held different opinions. Coutinho et al. reported that the exteriorized uterine repair had fewer operations exceeding 45 min total (56%) than with in situ repair (64.7%) [17]. Similarly, Ezechi et al. reported that the mean operative time was significantly less in the uterine exteriorization group (33.57 min) than in the in situ repair group (38.36 min). They concluded that shorter operating times also benefited from less blood loss and better accessibility while repairing the exteriorized uterus [34]. Moreover, in another study, a shorter time for uterine closure was found in the exteriorized uterine repair group than in the in situ group [47]. However, Doganay et al. reported significantly shorter mean operative time in the in situ uterine repair group (36.8 vs. 44.6 min) than in the exteriorized uterine repair group in a large-scale randomized controlled trial, including 5002 women [36]. They concluded the reason could have been the result of a more experienced operating team in the in situ uterine repair group. Although the exteriorization of the uterus requires time, it provides better exposure of the surgical field and good access for the surgeon to shorten the time for uterine repair. The methods of uterine repair can be selected by obstetricians according to their preference and the clinical situation. Certainly, further studies are warranted to clarify this issue.

#### 5.2.4. Uterine Atony

Peripartum hemorrhage has been a nightmare for obstetricians for centuries, and it accounts for about one-quarter to one-third of maternal deaths in the world [48]. One of the important causes of peripartum hemorrhage is uterine atony. Doganay et al. [36] compared the incidence of uterine atony under different repair techniques and found greater numbers of uterine atony in the exteriorized uterine repair group (9.1%) than the in situ repair group (3.8%). They assumed the result might be due to the longer operating time in their exteriorized repair group. Similarly, Abdellah et al. [44] reported a higher incidence of uterine atony and the need for additional uterotonics in the uterine exteriorization group. They also mentioned that the exteriorization of the uterus could increase the rate of uterine atony by a transient decrease in blood supply, but the event tended to disappear while repositioning the uterus into the abdominal cavity. Although these two studies observed an association between uterine atony and exteriorization for uterine repair during cesarean delivery, future studies are needed for further clarification of the relationship and its subsequent clinical impacts.

#### 5.2.5. Postoperative Complications

Different uterine repair techniques not only influenced the operative and maternal outcomes during cesarean delivery but also greatly impacted them after surgery. Postoperative pain could substantially relate to the patient’s satisfaction and subjective feelings about the surgery. Previous research has shown that patients who underwent exteriorized uterine repair during cesarean delivery had a greater chance to experience more severe pain and to take additional analgesia [17,46,47,49]. It is hypothesized that increased stretching of the uterine ligament and parietal peritoneum while exteriorizing the uterus contributed to this result [47]. 

Postpartum fever following cesarean delivery is a common obstetric complication, and it could be a sign of endometritis, urinary tract infection, wound infection, or phlebitis [50]. Therefore, detecting postpartum fever and determining the causes are important for obstetricians. Many studies investigated the rate of postoperative fever in different uterine repair groups, and most of the results revealed no significant differences among them [21,34,38,47,49,51]. Likewise, the incidences of endometritis and wound infection following cesarean delivery were similar in the two groups of exteriorized and in situ uterine repair techniques [17,34,35,36,37,44,47]. Batsu et al. [35] reported that manual removal of the placenta during cesarean surgeries is a major risk factor for endometritis. They did not find a significant difference in the incidence of endometritis between the two uterine repair techniques. Doganay et al. [36] noted a higher wound infection rate in the uterine exteriorization group but attributed it to the longer operation time in this group.

#### 5.2.6. Bowel Function

Ileus is one of the major problems after abdominal surgeries, along with increased hospital stays, postoperative pain, abdominal distension, and the inability to feed [52]. Certainly, cesarean delivery also carries a potential risk to cause such complications. Previous studies attempted to compare the return of bowel function between these two techniques of uterine repair. Most investigators concluded that the exteriorized uterine repair group needed more time for the return of bowel movements than the in situ (intra-abdominal) group [21,33,36,37,44,51]. Doganay et al. [36] reported that the in situ uterine repair group had sooner return of bowel sounds (mean time: 12 h) than the exteriorized uterine group (mean time: 14.9 h). El-Khayat et al. [37] stated similar results in a later study researching the exteriorized uterine repair group (mean time: 17 h) versus the in situ repair group (mean time: 14 h). Moreover, a recent meta-analysis yielded the same outcomes [21]. Since the bowels are manipulated less while performing in situ uterine repairs than exteriorized repairs, this phenomenon could explain such results. Although there was a significant difference in terms of the time to return of bowel movement between these two uterine repair techniques, further studies are needed to verify whether the use of different repair techniques is associated with the occurrence of postoperative ileus.

#### 5.2.7. Length of Hospitalization

The hospital stay often reflects the recovery status of the patients, and a longer hospital stay is usually associated with increased postoperative morbidity. In a literature review, several articles included the length of hospital stay as one of the outcome measures in different uterine repair groups. Orji et al. [33] reported significantly shorter hospitalization in the exteriorized uterine repair group (mean time: 6.7 days) than in the in situ (non-exteriorization) repair group (mean time: 7.6 days). However, in the study conducted by Doganay et al. [36], the mean hospital stay in the exteriorized uterine repair group was 2.6 days, which was longer than in the situ repair group (2.1 days). However, the difference did not reach a statistical significance (*p* > 0.05). A similar result was found in many previous studies and in a recent meta-analysis, which concluded that there was no significant difference between these two uterine repair techniques for the length of hospital stay [17,21,37,44,47]. In terms of the length of hospital stay, it seems that there is neither a marked difference nor a strong association by using different uterine repair techniques.

A summary of results in the literature was listed in Table 1 comparing the differences in the rates of intraoperative and postoperative complications and morbidities and the advantages and disadvantages of the techniques.

## 6. Discussion

Our analysis of the eligible articles in the literature reveals that vacuum assistance for fetal extraction is a simple, effective, and beneficial method of fetal head delivery. As an assistive method, vacuum assistance during cesarean section can facilitate the delivery of the fetal head and decrease the total blood loss. Nevertheless, vacuum assisted cesarean delivery possibly possesses a risk of fetal injuries even if the risk may be low. Compared with the in situ repair for a hysterotomy, the exteriorized uterine repair could have benefits including less blood loss and shorter operative time. However, it may also carry a higher risk of intraoperative complications such as nausea and vomiting, uterine atony, and a longer time for the return of bowel function. According to personal skills of training and gravidas’ conditions, obstetricians can take these points into consideration while deciding which assistive method to use and which surgical technique to perform.

In various clinical situations, the differences in using assistive methods and surgical techniques may have a significant impact. In planned or elective cesarean deliveries for term pregnancies, the choices of assistive methods and surgical techniques may depend on the surgeons’ training skills or preferences. However, in emergency or non-elective cesarean deliveries for term or preterm pregnancies with fetal distress, obstructive labor, severe preeclampsia, placenta abruption or other conditions, early delivery of the fetus and less blood loss are the major concerns. Therefore, vacuum assisted cesarean delivery and uterine exteriorization can be considered to facilitate the delivery of the fetus and the process of hemostasis. Theoretically, another advantage of applying uterine exteriorization to obstructed labor, which is an indication for non-elective cesarean delivery, is that the uterus is exteriorized and inspected to ensure there is no rupture on its surface. Furthermore, a proper intraoperative examination of pelvic adhesion and visualization of bilateral fallopian tubes are beneficial for subsequent pregnancy and delivery. Compared to first cesarean section, repeated elective cesarean delivery may carry a higher risk for pelvic adhesion. Thus, uterine exteriorization during the repeated surgery allows the surgeon to inspect directly on the peritoneum, uterus and tubes to ensure the conditions of the pelvic organs and local adhesion when the uterus is manipulated outside the abdominal cavity. Obstetricians should consider these factors in properly shared decision-making with pregnant women to decide on the most suitable technique for optimal cesarean delivery outcomes.

The strengths of the study include the source of databases, stricter screening methods, and inclusion criteria for selecting articles in the literature. Ovid Medline and PubMed, which we used for searching articles, are the largest databases in the literature review. Thus, the main findings and results of the topics would not be omitted. Another major advantage of the current review is stricter screening methods and inclusion criteria for selecting articles. Only full text articles were considered for inclusion to ensure that their contents can be inspected for quality control. Furthermore, two experts in the field reviewed the articles for the eligibility of inclusion after initial screening. Therefore, the results and inferences of the analysis from the retrieved articles would be robust and believable because of minimized possible errors that result from the searching process.

The current review has some inherent limitations despite its several strengths. The first limitation lies in the number of eligible articles in the field of surgical techniques and assistive methods used in cesarean deliveries. There were few eligible articles in both topics, thus limiting the application of the review result. Second, different results were reported with obstetricians of different skills and training, which may have affected the analysis in the current review. In addition, fewer randomized trials have been found in the topic of vacuum assisted cesarean delivery after selection. While all of the retrieved articles discussing uterine exteriorization vs. in situ repair were randomized trials, most of the retrieved articles discussing vacuum assisted cesarean delivery were not. Thus, the evidence available in vacuum assisted cesarean delivery is not as strong as that in uterine exteriorization. More details and comparisons among these surgical techniques and assistive methods are needed and warrant further investigation.

There exist some possible biases that may affect the results of the study. First, the selection bias may arise from the sources of databases, the searching strategies, the screening and excluding methods used for article selection in the review. Although the databases of Ovid Medline and PubMed we used for identifying articles are the largest two, a lack of searching other databases in the literature review can somewhat result in selection bias. Furthermore, the eligibility of selected articles was judged by two experts in the field. Despite the criteria set up for inclusion and exclusion, personal preferences during reviewing the articles can still bring about selection bias. Moreover, studies with positive findings have a higher chance to be published. There exists publication bias for the retrieved articles since studies with negative findings may be neglected.

## 7. Conclusions

Although several studies have recognized vacuum assistance for fetal extraction as a simple, effective, and beneficial method of fetal head delivery, further research is still required to clarify the safety of vacuum assistance in cesarean delivery. In general, exteriorized uterine repair could have benefits of less blood loss and shorter operative time compared to in situ uterine repairs. However, it may also carry a higher risk of intraoperative complications such as nausea and vomiting, uterine atony, and a longer time for the return of bowel function. Although these results have been observed in previous studies which focused on cesarean deliveries, whether these differences have a significant clinical impact still requires further investigation.

## Figures and Tables

**Table 1:** Summary of current studies regarding comparison two different techniques of uterine repair during cesarean delivery.

Authors Publish Year Country	Study Design	Population Age (Years)	Article Title	Main Results
**Blood Loss**				
Orji et al. [33] 2008 Nigeria	RCT	Mean ± SD	A randomized controlled trial of uterine exteriorization and non-exteriorization at cesarean section	Exteriorized uterine repair group had less blood loss than in situ uterine repair group
		Exteriorization 29.8 ± 6.6		
		Non-exteriorization 29.5 ± 6.1		
Ezechi et al. [34] 2005 Nigeria	RCT	Mean ± SD	Uterine incision closure at cesarean section: a randomized comparative study of intraperitoneal closure and closure after temporary exteriorization	Exteriorized uterine repair group had less blood loss than in situ uterine repair group
		In situ 29.1 ± 4.5		
		Exteriorization 29.3 ± 4.8		
Baksu et al. [35] 2005 Turkey	Randomized clinical trial	Mean ± SD	The effect of placental removal method and site of uterine repair on postcesarean endometritis and operative blood loss	The study did not report significant differences in blood loss between exteriorized uterine repair group and in situ uterine repair group
		In situ (2 subgroups) 22.9 ± 7.2; 25.2 ± 4.3		
		Exteriorization (2 subgroups) 23.4 ± 4.1; 24.6 ± 5.4		
**Intraoperative Complications**				
Siddiqui et al. [42] 2008 Canada	RCT	Similar between two groups	Complications of exteriorized compared with in situ uterine repair at cesarean delivery under spinal anesthesia: a randomized controlled trial	Higher incidence of intraoperative nausea and vomiting in exteriorized uterine repair group than in situ uterine repair group
Abdellah et al. [44] 2018 Egypt	Randomized clinical trial	Mean ± SD	Uterine exteriorization versus intraperitoneal repair: effect on intraoperative nausea and vomiting during repeat cesarean delivery—A randomized clinical trial	Higher incidence of intraoperative nausea and vomiting in exteriorized uterine repair group than in situ uterine repair group
		Intraperitoneal repair 27.67 ± 5.22		
		Uterine exteriorization 28.34 ± 5.44		
**Operative Time**				
Coutinho et al. [17] 2008 Brazil	RCT	Mean ± SD	Uterine exteriorization compared with in situ repair at cesarean delivery: a randomized controlled trial	Exteriorized uterine repair group had fewer operations that exceeded 45 min than in the in situ repair group
		In situ 25.6 ± 6.3		
		Exteriorized uterus 24.7 ± 6.1		
Walsh et al. [22] 2009 UK	Meta-analysis		Extra-abdominal vs. intra-abdominal uterine repair at cesarean delivery: a meta-analysis	No significant difference in operative time between in situ uterine repair and exteriorized uterine repair groups
Orji et al. [33] 2008 Nigeria	RCT	Mean ± SD	A randomized controlled trial of uterine exteriorization and non-exteriorization at cesarean section	No significant difference in operative time between in situ uterine repair and exteriorized uterine repair groups
		Exteriorization 29.8 ± 6.6		
		Non-exteriorization 29.5 ± 6.1		
Ezechi et al. [34] 2005 Nigeria	RCT	Mean ± SD	Uterine incision closure at cesarean section: a randomized comparative study of intraperitoneal closure and closure after temporary exteriorization	Mean operative time was less in uterine exteriorization group than in the in situ repair group
		In situ 29.1 ± 4.5		
		Exteriorization 29.3 ± 4.8		
Doganay et al. [36] 2010 Turkey	RCT	Mean ± SD	Effects of method of uterine repair on surgical outcome of cesarean delivery.	Significantly shorter mean operative time in the in situ uterine repair group than in the exteriorized uterine repair group
		In situ 31.76 ± 2.59		
		Exteriorized uterus 32.66 ± 1.87		
Wahab et al. [38] 2005 United Kingdom	RCT	Mean ± SD	A randomized, controlled study of uterine exteriorization and repair at cesarean section	No significant difference in operative time between in situ uterine repair and exteriorized uterine repair groups
		Exteriorized No recorded		
		Non-exteriorized No recorded		
Siddiqui et al. [42] 2007 Canada.	RCT	Mean ± SD	Complications of exteriorized compared with in situ uterine repair at cesarean delivery under spinal anesthesia: a randomized controlled trial	No significant difference in operative time between in situ uterine repair and exteriorized uterine repair groups
		In situ 34.1 ± 5.1		
		Exteriorized uterus 33.0 ± 4.7		
Edi-Osagie et al. [43] 1998 UK	RCT	Mean ± SD	Uterine exteriorization at cesarean section: influence on maternal morbidity	No significant difference in operative time between in situ uterine repair and exteriorized uterine repair groups
		Exteriorization 28.01± 5.71		
		In situ repair 29.02 ± 6.29		
Mohr-Sasson et al. [46] 2020 Israel	RCT	Mean (Range)	Uterine exteriorization versus intraperitoneal repair in primary and repeat cesarean delivery: a randomized controlled trial	No significant difference in operative time between in situ uterine repair and exteriorized uterine repair groups
		Intraperitoneal 35 (31–38)		
		Extra-abdominal 34 (30–37)		
Chauhan et al. [47] 2018 India	RCT	Mean ± SD	A randomized comparative study of exteriorization of uterus versus in situ intraperitoneal repair at cesarean delivery	Shorter uterine closure time in exteriorized uterine repair group than in situ uterine repair group
		Extra abdominal repair 27.42 ± 3.71		
		In situ Repair 27 ± 3.82		
**Uterine Atony**				
Doganay et al. [36] 2010 Turkey	RCT	Mean ± SD	Effects of method of uterine repair on surgical outcome of cesarean delivery	Higher rate of uterine atony in exteriorized uterine repair group than in the in situ repair group
		In situ 31.76 ± 2.59		
		Exteriorized uterus 32.66 ± 1.87		
Abdellah et al. [44] 2018 Egypt	Randomized clinical trial	Mean ± SD	Uterine exteriorization versus intraperitoneal repair: effect on intraoperative nausea and vomiting during repeat cesarean delivery—A randomized clinical trial	Higher incidence of uterine atony and need for uterotonics in exteriorization group than in situ uterine repair group
		Intraperitoneal repair 27.67 ± 5.22		
		Uterine exteriorization 28.34 ± 5.44		
**Length of Hospitalization**				
Coutinho et al. [17] 2008 Brazil	RCT	Mean ± SD	Uterine exteriorization compared with in situ repair at cesarean delivery: a randomized controlled trial	No significant difference of length of hospitalization stay in situ uterine repair and exteriorized uterine repair groups
		In situ 25.6 ± 6.3		
		Exteriorized uterus 24.7 ± 6.1		
Orji et al. [33] 2008 Nigeria	RCT	Mean ± SD	A randomized controlled trial of uterine exteriorization and non-exteriorization at cesarean section	Significantly shorter hospitalization in exteriorized uterine repair group than in situ uterine repair group
		Exteriorization 29.8 ± 6.6		
		Non-exteriorization 29.5 ± 6.1		
Doganay et al. [36] 2010 Turkey	RCT	Mean ± SD	Effects of method of uterine repair on surgical outcome of cesarean delivery	Mean hospital stays in exteriorization uterine repair group was 2.6 days longer than in situ repair group (2.1 days), but which did not reach the statistical significance (*p* > 0.05).
		In situ 31.76 ± 2.59		
		Exteriorized uterus 32.66 ± 1.87		
El-Khayat et al. [37] 2014 Egypt	RCT	Mean ± SD	A randomized controlled trial of uterine exteriorization versus in situ repair of the uterine incision during cesarean delivery	No significant difference in length of hospitalization stay between in situ uterine repair and exteriorized uterine repair groups
		Extra-abdominal repair 27.1 ± 1.9		
		In situ repair 27.0 ± 2.0		
Abdellah et al. [44] 2018 Egypt	Randomized clinical trial	Mean ± SD	Uterine exteriorization versus intraperitoneal repair: effect on intraoperative nausea and vomiting during repeat cesarean delivery—A randomized clinical trial	No significant difference in length of hospitalization stay between in situ uterine repair and exteriorized uterine repair groups
		Intraperitoneal repair 27.67 ± 5.22		
		Uterine exteriorization 28.34 ± 5.44		
Chauhan et al. [47] 2018 India	RCT	Mean ± SD	A randomized comparative study of exteriorization of uterus versus in situ intraperitoneal repair at cesarean delivery	No significant difference in length of hospitalization stay between in situ uterine repair and exteriorized uterine repair groups
		Extra abdominal Repair 27.42 ± 3.71		
		In situ Repair 27 ± 3.82		

RCT: randomized controlled trial.

## References

[1] World Health Organization Human Reproduction Programme (2015). WHO Statement on Caesarean Section Rates. Rep. Health Matters.

[2] Betrán A.P., Merialdi M., Lauer J.A., Thomas J., Van Look P., Wagner M., Bing-Shun W. (2007). Rates of Caesarean Section: Analysis of Global, Regional and National Estimates. Paediatr. Peérinat. Epidemiology.

[3] Barber E.L., Lundsberg L.S., Belanger K., Pettker C.M., Funai E.F., Illuzzi J.L. (2011). Indications Contributing to the Increasing Cesarean Delivery Rate. Obstet. Gynecol..

[4] Li H.-T., Luo S., Trasande L., Hellerstein S., Kang C., Li J.-X., Zhang Y., Liu J.-M., Blustein J. (2017). Geographic Variations and Temporal Trends in Cesarean Delivery Rates in China, 2008-2014. JAMA.

[5] Mittal S., Pardeshi S., Mayadeo N., Mane J. (2014). Trends in Cesarean Delivery: Rate and Indications. J. Obstet. Gynecol. India.

[6] Martin J.A., E Hamilton B., Osterman M.J.K. (2014). Births in the United States, 2013. NCHS Data Brief.

[7] Villar J., Valladares E., Wojdyla D., Zavaleta N., Carroli G., Velazco A., Shah A., Campodonico L., Bataglia V., Faundes A. (2006). Caesarean Delivery Rates and Pregnancy Outcomes: The 2005 WHO Global Survey on Maternal and Perinatal Health in Latin America. Lancet.

[8] Delport S. (2019). Global Epidemiology of Use of and Disparities in Caesarean Sections. Lancet.

[9] Encarnacion B., Zlatnik M.G. (2012). Cesarean Delivery Technique. Obstet. Gynecol. Surv..

[10] Jacobs-Jokhan D., Hofmeyr G.J. (2004). Extra-Abdominal Versus Intra-Abdominal Repair of the Uterine Incision at Caesarean Section. Cochrane Database Syst. Rev..

[11] Bamigboye A.A., Hofmeyr G.J. (2014). Closure Versus Non-Closure of the Peritoneum at Caesarean Section: Short and Long-Term Outcomes. Cochrane Database Syst. Rev..

[12] Gates S. (2004). Techniques and Materials for Closure of the Abdominal Wall in Caesarean Section. Cochrane Database Syst. Rev..

[13] Alderdice F., McKenna D., Dornan J. (2003). Techniques and Materials for Skin Closure in Caesarean Section. Cochrane Database Syst. Rev..

[14] Mathai M., Hofmeyr G.J., Mathai N.E. (2013). Abdominal Surgical Incisions for Caesarean Section. Cochrane Database Syst. Rev..

[15] Seiler C.M., Deckert A., Diener M.K., Knaebel H.-P., Weigand M.A., Victor N., Büchler M.W. (2009). Midline Versus Transverse Incision in Major Abdominal Surgery. Ann. Surg..

[16] Hofmeyr J.G., Novikova N., Mathai M., Shah A. (2009). Techniques for Cesarean Section. Am. J. Obstet. Gynecol..

[17] Coutinho I.C., Amorim M.M., Katz L., De Ferraz A.A.B. (2008). Uterine Exteriorization Compared With In Situ Repair at Cesarean Delivery: A Randomized Controlled Trial. Obstet. Gynecol..

[18] Minkoff H., Chervenak F.A. (2003). Elective Primary Cesarean Delivery. N. Engl. J. Med..

[19] Penzias A., Bendikson K., Falcone T., Gitlin S., Gracia C., Hansen K., Hill M., Hurd W., Jindal S., Kalra S. (2019). Postoperative Adhesions in Gynecologic Surgery: A Committee Opinion. Fertil. Steril..

[20] Diamond M.P., Freeman M.L. (2001). Clinical Implications of Postsurgical Adhesions. Hum. Reprod. Updat..

[21] Zaphiratos V., George R.B., Boyd J.C., Habib A. (2015). Uterine Exteriorization Compared with In Situ Repair for Cesarean Delivery: A Systematic Review and Meta-Analysis. Canad. J. Anesthesia/J. Canadien D’anesthésie.

[22] Walsh C.A., Walsh S.R. (2009). Extraabdominal vs Intraabdominal Uterine Repair at Cesarean Delivery: A Metaanalysis. Am. J. Obstet. Gynecol..

[23] Waterfall H., Grivell R., Dodd J.M. (2016). Techniques for Assisting Difficult Delivery at Caesarean Section. Cochrane Database Syst. Rev..

[24] Johanson R., Menon V. (2000). Soft Cersus Rigid Vacuum Extractor Cups for Assisted Vaginal Delivery. Cochrane Database Syst Rev.

[25] Solomons E. (1962). Delivery of the Head with the Malmstrom Vacuum Extractor During Cesarean Section. Obstet. Gynecol..

[26] McQuivey R.W., E Block J. (2017). Vacuum-Assisted Cesarean Section. Int. J. Women’s Heal..

[27] Bofill J.A., Lencki S.G., Barhan S., Ezenagu L.C. (2000). Instrumental Delivery of the Fetal Head at the Time of Elective Repeat Cesarean: A Randomized Pilot Study. Am. J. Perinatol..

[28] Dimitrov A., Pavlova E., Krŭsteva K., Nikolov A. (2008). Caesarean Section with Vacuum Extraction of the Head. Akush. Ginekol..

[29] Clark S.L., Vines V.L., Belfort M.A. (2008). Fetal Injury Associated with Routine Vacuum Use during Cesarean Delivery. Am. J. Obstet. Gynecol..

[30] Arad I., Linder N., Bercovici B. (1986). Vacuum Extraction at Cesarean Section—Neonatal outcome. J. Périnat. Med..

[31] Sukit Sritippayawan W.C. (2011). Assisted Delivery of High Floating Fetal Head: A Comparison of Vacuum-Assisted Delivery with Manual Extraction. Asian Biomed..

[32] Pearson G.A., MacKenzie I. (2014). Blood Loss and Blood Transfusion at Caesarean Section: A Prospective Observational Study Covering 30 Years. Eur. J. Obstet. Gynecol. Reprod. Boil..

[33] Orji E.O., Olaleye A.O., Loto O.M., Ogunniyi S.O. (2008). A Randomised Controlled Trial of Uterine Exteriorisation and Non-Exteriorisation at Caesarean Section. Aust. N. Z. J. Obstet. Gynaecol..

[34] Ezechi O.C., E Kalu B.K., O Njokanma F., A Nwokoro C., E Okeke G.C. (2005). Uterine Incision Closure at Caesarean Section: A Randomised Comparative Study of Intraperitoneal Closure and Closure after Temporary Exteriorisation. West Afr. J. Med..

[35] Baksu A., Kalan A., Ozkan A., Baksu B., Tekelioglu M., Goker N. (2005). The Effect of Placental Removal Method and Site of Uterine Repair on Postcesarean Endometritis and Operative Blood Loss. Acta Obst. Gyn. Scand..

[36] Doğanay M., Tonguc E.A., Var T. (2010). Effects of Method of Uterine Repair on Surgical Outcome of Cesarean Delivery. Int. J. Gynecol. Obstet..

[37] El-Khayat W., ElSharkawi M., Hassan A. (2014). A Randomized Controlled Trial of Uterine Exteriorization Versus In Situ Repair of the Uterine Incision During Cesarean Delivery. Int. J. Gynecol. Obstet..

[38] Wahab M.A., Karantzis P., Eccersley P.S., Russell I.F., Thompson J.W., Lindow S.W. (1999). A Randomised, Controlled Study of Uterine Exteriorization and Repair at Caesarean Section. BJOG Int. J. Obstet. Gynaecol..

[39] Abalos E., Addo V., Brocklehurst P., El Sheikh M., Farrell B., Gray S., Hardy P., Juszczak E., E Mathews J., Masood S.N. (2013). Caesarean Section Surgical Techniques (CORONIS): A Fractional, Factorial, Unmasked, Randomised Controlled Trial. Lancet.

[40] Shin D.W., Kim Y., Hong B., Yoon S.-H., Lim C.S., Youn S. (2019). Effect of Fentanyl on Nausea and Vomiting in Cesarean Section Under Spinal Anesthesia: A Randomized Controlled Study. J. Int. Med Res..

[41] Mishriky B.M., Habib A. (2012). Metoclopramide for Nausea and Vomiting Prophylaxis During and After Caesarean Delivery: A Systematic Review and Meta-Analysis. Br. J. Anaesth..

[42] Siddiqui M., Goldszmidt E., Fallah S., Kingdom J., Windrim R., Carvalho J.C.A. (2007). Complications of Exteriorized Compared with In Situ Uterine Repair at Cesarean Delivery Under Spinal Anesthesia. Obstet. Gynecol..

[43] Edi-Osagie E.C.O., Hopkins R.E., Ogbo V., Lockhat-Clegg F., Ayeko M., Akpala W.O., Mayers F.N. (1998). Uterine Exteriorisation at Caesarean Section: Influence on Maternal Morbidity. Br. J. Obstet. Gynaecol..

[44] Abdellah M.S., Abbas A.M., Ali M.K., Mahmoud A., A Abdullah S. (2018). Uterine Exteriorization Versus Intraperitoneal Repair: Effect on Intraoperative Nausea and Vomiting During Repeat Cesarean Delivery𢀔A Randomized Clinical Trial. Facts Views Vis. ObGyn.

[45] Mireault D., Loubert C., Drolet P., Tordjman L., Godin N., Richebé P., Zaphiratos V. (2020). Uterine Exteriorization Compared With In Situ Repair of Hysterotomy After Cesarean Delivery. Obstet. Gynecol..

[46] Mohr-Sasson A., Castel E., Lurie I., Heifetz S., Kees S., Sivan E. (2020). Uterine Exteriorization Versus Intraperitoneal Repair in Primary and Repeat Cesarean Delivery: A Randomized Controlled Trial. J. Matern. Neonatal Med..

[47] Chauhan S. (2018). A Randomized Comparative Study of Exteriorization of Uterus Versus In Situ Intra-Peritoneal Repair at Cesarean Delivery. Int. J. Reprod. Contracept. Obstet. Gynecol..

[48] Weeks A.D. (2014). The Prevention and Treatment of Postpartum Haemorrhage: What Do We Know, and Where Do We Go to Next?. BJOG Int. J. Obstet. Gynaecol..

[49] Nafisi S. (2007). Influence of Uterine Exteriorization Versus In Situ Repair on Post-Cesarean Maternal Pain: A Randomized Trial. Int. J. Obstet. Anesthesia.

[50] Hamadeh G., Dedmon C., Mozley P.D. (1995). Postpartum Fever. Am. Fam. Physician.

[51] Özbay K. (2011). Exteriorized Versus In-Situ Repair of the Uterine Incision at Cesarean Delivery: A Randomized Controlled Trial. Clin. Exp. Obstet. Gynecol..

[52] Mohsenzadeh-Ledari F., Barat S., Delavar M.A., Banihosini S.Z., Khafri S. (2013). Chewing Sugar-Free Gum Reduces Ileus after Cesarean Section in Nulliparous Women: A Randomized Clinical Trial. Iran. Red Crescent Med. J..

